# Feasibility of high-throughput drug sensitivity screening (HDS)-guided treatment for children with refractory or relapsed acute myeloid leukemia

**DOI:** 10.3389/fped.2023.1117988

**Published:** 2023-02-17

**Authors:** Wenxiu Lv, Tianping Chen, Shen Wang, Chun Li, Bo Zhang, Liang Wang, Fang Xv, Fang Cao, Jing Wang, Li Chen, Chenglin Liao, Na Li, Hongjun Liu

**Affiliations:** ^1^ Department of Hematology and Oncology, Anhui Provincial Children's Hospital (Anhui Hospital, Pediatric Hospital of Fudan University), Hefei, Anhui, China; ^2^ Department of Pediatrics, The First Affiliated Hospital of USTC, Division of Life Sciences and Medicine, University of Science and Technology of China, Hefei, Anhui, China

**Keywords:** pediatric, high-throughput drug sensitivity screening, hematopoietic stem cell transplantation (HCST), relapsed/refractory, acute myeloid leukemia

## Abstract

Relapsed/refractory (rel/ref) acute myeloid leukemia (AML) has a very high mortality rate. At present, hematopoietic stem cell transplantation (HSCT) is the most effective treatment for rel/ref AML. The remission of the primary disease before HSCT is crucial for the transplantation to be effective. Therefore, it is critical to choose a suitable type of chemotherapy before HSCT. Here, we recorded the outcomes of high-throughput drug sensitivity screening (HDS) in children with rel/ref AML. Thirty-seven pediatric rel/ref AML patients who received HDS from September 2017 until July 2021 were analyzed retrospectively. Most of the patients (24 patients, 64.9%) had adverse cytogenetics. Two patients had rel/ref AML with central nervous system leukemia. The complete remission (CR) rate was 67.6%. Eight patients developed IV grade bone marrow suppression. Twenty-three patients (62.2%) underwent HSCT. The 3-year overall survival (OS) and EFS rates were 45.9% and 43.2%, respectively. Infection in the myelosuppression stage was the main cause of death. The outcome of HDS was superior to the commonly reported rates. These results suggest that HDS may be a novel treatment option for pediatric patients with rel/ref AML, and it is a promising transitional regimen prior to HSCT.

## Introduction

Acute myeloid leukemia (AML) is a clinically, morphologically, and genetically heterogeneous disorder (1) and is relatively rare in children. With rapid advances in the study and treatment of pediatric AML, the cure rates of pediatric AML have significantly improved. The overall survival rate (OS) of AML in children is more than 60% (2–4). However, the long-term disease-free survival rate is only approximately 40% after the administration of standard chemotherapy (5). Some children eventually progress to relapsed/refractory (rel/ref) AML. Once rel/ref AML occurs, there is a high increase in the mortality rate. It is generally believed that children with rel/ref AML have little chance of being cured through chemotherapy alone (6, 7). Hematopoietic stem cell transplantation (HSCT) is one approach for the treatment of rel/ref AML (8–11). Induction therapy before HSCT is critical for children with rel/ref AML.

The therapeutic efficacy of HSCT depends on whether the patient has achieved complete remission (CR) before HSCT, and many centers also carry out tentative treatment for rel/ref AML (12, 13). At present, numerous tumor cell targets have been found, and targeted therapy is increasingly favored by researchers. However, it is difficult to obtain the sensitivity data of anticancer drugs. High-throughput drug sensitivity screening (HDS) is a drug sensitivity detection technology for regenerative primary cancer cells. In recent years, many researchers have carried out clinical research on new primary cancer cell culture technologies (14). Tyner et al. reported that drug sensitivity tests could be used to guide the clinical medication of AML (15). However, the data on HDS for patients with rel/ref AML are scarce. Our study cohort includes the outcomes of 37 consecutive children with rel/ref AML who received HDS at our center from September 2017 to July 2021. HDS is expected to find treatments that may help improve the clinical outcomes of rel/ref pediatric AML.

## Patients and methods

### Selection of patients

In this study, we reviewed the medical records of 37 consecutive children with rel/ref AML who received HDS at the first Affiliated Hospital of the University of Science and Technology of China from September 2017 to July 2021. All patients with newly diagnosed rel/ref AML were younger than 14 years of age. The diagnosis was based on the published criteria (16). The choice of frontline treatment for HDS was made by the patients and their guardians, from whom informed consent was obtained according to the Declaration of Helsinki. The study was approved by the Ethics Committee of the first Affiliated Hospital of the University of Science and Technology of China.

### HDS therapy

After the patients enrolled themselves, their guardians signed the informed consent form. Before the initiation of treatment, the liver and kidney functions of the patients were examined to avoid contraindication of therapy. Bone marrow was collected under an aseptic condition, placed in an EDTA anticoagulant tube, refrigerated at 2 °C – 8 °C, and transported to *PreceDo Inc.* (Hefei, China). The primary tumor cells isolated and purified according to the standard operating procedure were expanded in vitro by an improved cell reprogramming technique. The cultured primary cells were tested for high-throughput drugs in vitro according to the clinical first-line and second-line treatment schemes of the corresponding cancer types and FDA drug bank, and the sensitive drugs and schemes were selected. The experiment was performed according to the procedure laid down in a previous report (17). The growth inhibition rates of different chemotherapeutic drugs were calculated in the laboratory, and test reports were prepared in the clinic. In contrast to the reference, the drug inhibition rates were classified as follows: high sensitivity (+++): inhibition rate ≥80%; moderate sensitivity (++): inhibition rate of 50%–80%; and low sensitivity (+): inhibition rate of 20%–<50%. After receiving the test report, the department selected the chemotherapy plan according to the test report. After the start of chemotherapy, the adverse effects on the patients were recorded. Three days after the end of the treatment course, the cardiac B-ultrasound, electrocardiogram, and biochemical indices were reexamined to observe the level of toxicity of the drug. The myelograms and peripheral blood routine were reexamined 14 days after the course of treatment; morphology, immunophenotype, cytogenetics, and molecular biology classification were performed, and the morphological characteristics of the cells were analyzed. When any signs of fungal infection were observed, antifungal drugs such as fluconazole/voriconazole and echinocandins were added to the treatment protocol.

### Statistical analysis

The probable OS was defined as the time of relapse diagnosis to the date of the last follow-up or death. The event-free survival (EFS) was defined as “patient-censored second events.” Events included death, second relapse, second malignancy (SMN), death caused by resistant disease or reinduction failure, or death by undefined causes.

To assess the effect of treatment on patient outcomes, the probabilities of OS and EFS were assessed. The Kaplan–Meier method was applied for generating survival curves for EFS and OS. *χ*^2^ were used to compare the differences in the distributions of different parameters. The Mann–Whitney test was used for making a comparison between groups, which generates non-normal distribution data. The SPSS25 statistical program was used for statistical analysis.

## Results

### Patient characteristics

**Figure 1:**
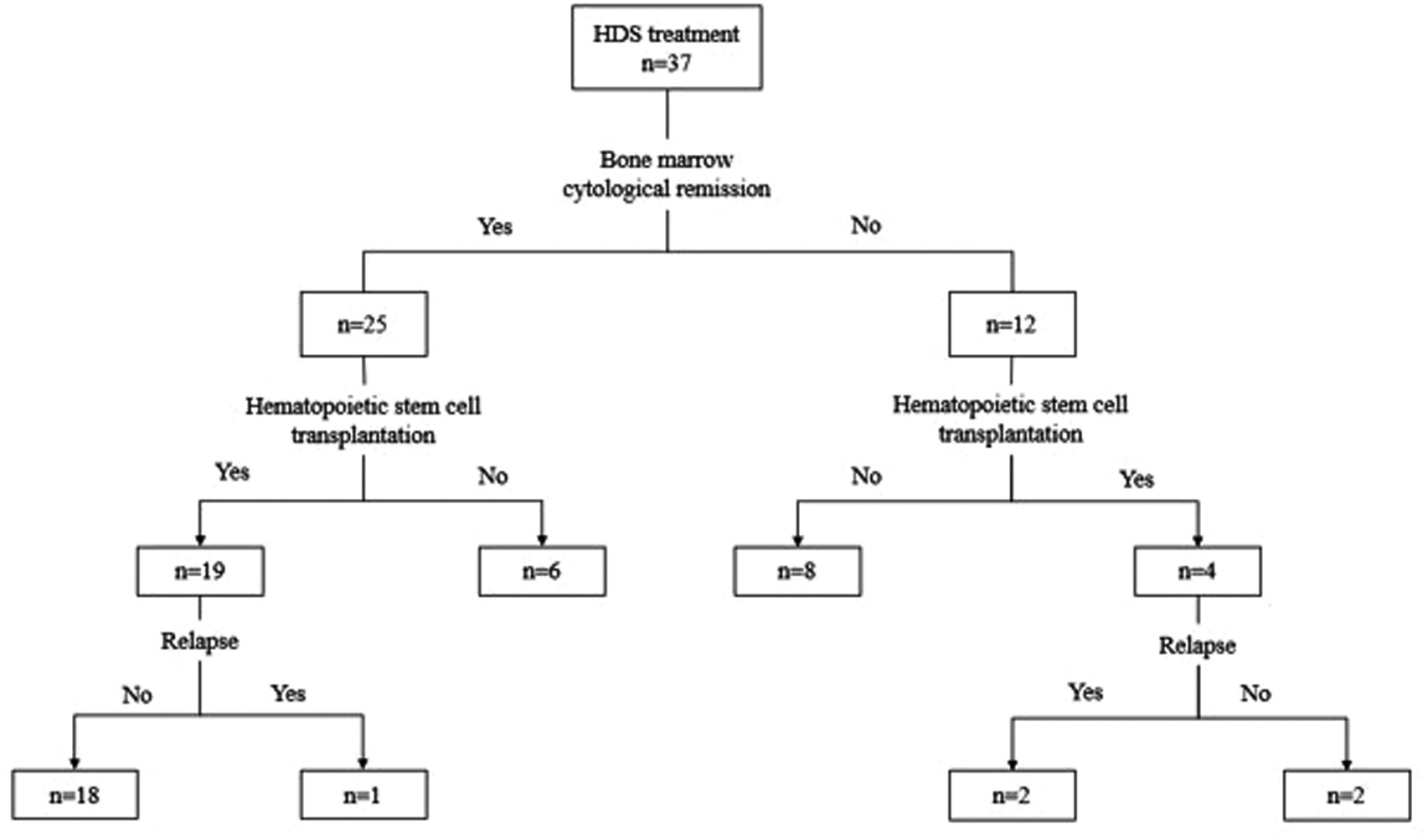
Treatment flows and outcomes for 37 patients.

Between 2017 and 2021, a total of 37 patients with rel/ref AML were enrolled in this study. The previous treatment of primary AML was performed according to the procedure described in a previous report (18). The overall treatment flow and outcomes of patients in this study are shown in Figure 1. The characteristics of the 37 children are presented in Table 1, which include age, sex, classification subtype, genetic anomaly at diagnosis, and time from initial diagnosis of AML to relapse. The median age of patients was 5 years (range, 1–15 years). In line with WHO classification (16), 2 patients were diagnosed with M1, 13 with M2, 4 with M4, 12 with M5, 1 with M6, and 5 with M7. All patients were assessed for genetic abnormalities before treatment. Thirteen patients had a normal karyotype without any molecular abnormality, six had *t* (8 : 21) mutations, eight had *AML1-ETO* mutations, and all patients with *t* (8 : 21) chromosome abnormalities tested positive for the *AML1-ETO* gene. One patient had *mixed lineage leukemia-AF9* mutations, 3/37 (8.1%) patients tested positive for the *WT1-ABL* gene, 2/37 (5.4%) were positive for the *CEBPA* gene, 2/37 (5.4%) were positive for the *FLT-ITD* gene, 2/37 (5.4%) were positive for the *CBFβ/MYH11* gene, and 1/37 (2.7%) patient was positive for the *BCR-ABL* gene. Two patients tested positive for the *TET2* gene, including one with a positive double mutation of the *CEBPA* gene. One patient was positive for the *EVI1* gene, 1/37 (2.7%) was positive for the *NRAS* gene, and 1/37 (2.7%) was positive for the *NuP98/KDM5A* gene. Thirty-one patients had relapsed AML and six patients had refractory AML. Of all children with relapsed AML, only two children relapsed longer than 18 months. Two patients had central nervous system leukemia.

**Table 1:** Baseline characteristics of the patients.

	No. (%)
**Age**	
<10 years	26 (70.3)
≥10 years	11 (29.7)
**Gender**	
M	18 (48.6)
F	19 (51.4)
**WHO classification subtype**	
M1	2 (5.4)
M2	13 (35.1)
M3	0
M4	4 (10.8)
M5	12 (32.4)
M6	1 (2.7)
M7	5 (13.5)
**Genetic anomaly at diagnosis**	
Normal karyotype without any molecular abnormality	13 (35.1)
*t* (8 : 21)	6 (13.5)
*AML1-ETO*^a^	8 (21.6)
*Mixed lineage leukemia-AF9*	1 (2.7)
*WT1-ABL*	3 (8.1)
*CEBPA*	2 (5.4)
*FLT-ITD*	2 (5.4)
*CBFβ/MYH11*	2 (5.4)
*BCR-ABL*	1 (2.7)
*TET2*^b^	2 (5.4)
*EVI1*	1 (2.7)
*NRAS*	1 (2.7)
*NuP98/KDM5A*	1 (2.7)
**Disease status**	
Relapsed	31 (81.6)
Refractory	6 (16.2)
**Recurrence time** ^c^	
≤18months	29 (93.5)
>18months	2 (6.5)

^a^ All patients with *t* (8 : 21) chromosome abnormalities tested positive for the *AML1-ETO* gene.

^b^ One patient tested positive for the double mutation of the *CEBPA* gene.

^c^ The total number of relapses was 31.

### Treatment outcome

The HDS results of each patient are summarized in Table 2. Clinicians choose appropriate chemotherapy schemes according to the sensitivity of tumor cells to different chemotherapy schemes. Twenty-five patients who received treatment in our department responded to the treatment modalities, with a CR rate of 67.6%. The patients were divided into different subgroups to analyze the relationship between CR and various factors. The responses to treatment modalities are shown in Table 3. Age and sex had no significant influence on the treatment results. There was no statistical significance of classification subtype (*P* = 0.655) or genetic anomaly at diagnostic plays (*P* = 0.326). The majority of the 37 patients included in the study had relapsed AML, and there was no significant difference in the disease status (*P* = 0.367). Of the two patients with late recurrence, one had CR, and early recurrence showed no significant effect on the outcome of treatment (*P* = 0.499). The treatment of the central nervous system involves multiple three intrathecal chemotherapy, and none of them achieved CR.

**Table 2:** HDS results for each patient.

No.	Sensitivity			Chosen treatment regimen	Whether achieved CR
	High	Moderate	Low		
1	IA	DAC/MA/HAD/HD-DA	DA/DAT/HAA/HA	DAC + HAD	Y
2	—	—	HOAP/DAT/HA/ME	HOAP	N
				HA	N
3^a^	—	DAT/HAD/HD-DA/HOAP	DAC/EA/DA/MA/IA	DAC + HAA	Y
				DAC + HAA	Y
4	—	MA/HAD	DAC/EA/HD-DA	DAC + HAD	Y
5	HOAP/HA/HAD/ME/HD-DA	MA	—	HAD	Y
6	—	IAE/DAE/FLAG/CLAG	DCAG/ME/HOAP/HD-DA/DAT	DAC + HAA	Y
7	—	HAA/HOAP/DAT/DAE	HA2/HD-DA/ME	DAC + HAA	Y
8	—	HA/HAA/HAD	IAE/DAC/DAT	DAC + HAA	Y
9	—	HAA/HA/MA	DAC/EA/IA	DAC + HAA	Y
10	—	—	IA/HAD/MA/HOAP	HAD	N
				IA	N
11	—	HAA/HAD/MA	DAC/DA	DAC + HAA	Y
12		HAD/HA	EA/IA/MA	HAD	Y
13	HAD	HAD + VP-16/VDLP	VCR + ADM	HAD	Y
14	—	—	CAM/HA	HAA	N
				CAM	N
15	—	—	HAD/HA/EA	HAA	N
				HAD	N
16	—	HAD/HA	DAC/IEA/DA	DAC + HAD	Y
17	—	—	HAD/HD-DA/DAC	HAD	N
18	HAA/HOAP/HD-HA	DAC/DCAG	—	DAC + HAA	Y
19	—	DAE/IAE	DAC/HA/IA	DAE	N
				IAE	N
				DAC + HAA	N
				DAC + HAA	N
20	FLAG/IA	DAC	MA	DAC + IA	N
				FLAG	N
21	FLAG/HD-Arac	HAA/IAE/MOAP	—	FLAG	N
				DAC + HAA	N
22^b^	—	DCAG/HD-DA	IAE/CLAG/HOAP/FLAG	DCAG	Y
23	—	DAE/CLAG/FLAG	HOAP/MA/CAG/ME	DAE	Y
24	IA/DAC/HAD	HD-DA	ME/HA/DA/MA1	DAE	Y
25^c^	DAC/HD-DA/HAD	DAT/HAD/HD-DA/HOAP	HAA/HOAP	DAC + HAD	Y
				DAC + HAA	N
26	—	IAE/DAC/DAE/CAG	DA/HD-DA/ME/HOAP/HA	DAC + CLAG	Y
27	—	DAC/HAD/DA	IAE/IA/EA	DAC + HAD	N
28^d^	—	DA/IA/HA	MA/EA/ME	IA	N
				DAC + HAA	N
29	HAA/FLAG/HA	DAC/DAT	—	FLAG	Y
30	IDAG/HAD/DAC	HD-DA/HOAP	MA/EA	DAC + IDAG	Y
31	—	IDAG/HAD/HAA	IA/CAG/HOAP	DAC + IDAG	Y
32	—	HAA/HAD/COAD	CLAG/IDAD/DA	HAD	Y
33	—	HAD/COAD/ID	DA/EA/FLAG	COAD	N
				HAD	N
34	CLAG/HAA/HAD	DA/HA/HOAP	MA/CAG	DAC + HAA	N
				CLAG	Y
35	—	HA/DA	MA/IA/EA	HAA	N
36	IDAG/HAD	DAC/HA	—	DAC + IDAG	Y
37	IDAD/HAA/CLAG	ABT199 + HA/CAM	MA/COAD	DAC + IDAG	N
				IDAD	N
				ABT199 + HD-Arsc	Y

DAC, 5-aza-2-deoxycytidine; HAA, homoharringtonine + cytarabine + aclarubicin; HAD, homoharringtonine + cytarabine + daunorubicin; HA, homoharringtonine + cytarabine; VP-16, etoposide; ABT199, venetoclax; IA, idarubicin + cytarabine; MA, mitozantrone + cytarabine; DA, daunorubicin + cytarabine; HD-DA, daunorubicin + cytarabine 300 mg/(m^2^ d); HD-HA, homoharringtonine + cytarabine 300 mg/(m^2^ d); EA, etoposide + cytarabine; DAE, daunorubicin + cytarabine + etoposide; HOAP, harringtonine + vincristine + cytarabine + prednisone; HD-Arac, cytarabine 300 mg/(m^2^ d); CLAG, cladribine + cytarabine + granulocyte-colony stimulating factor; FLAG, fludarabine + cytarabine + granulocyte-colony stimulating factor; ME, mitozantrone + etoposide; CAM, cyclophosphamide + cytarabine + mercaptopurine; DAT, daunorubicin + cytarabine + 6-thioguanine; DCAG, deoxycytidine + arubicin + cytarabine + granulocyte-colony stimulating factor; IAE, cytarabine + etoposide + idarubicin; CAG, cytarabine + aclarubicin + granulocyte-colony stimulating factor; MOAP, mitoxantrone + vincristine + cytarabine + prednisone; COAD, cyclophosphamide + vincristine + cytarabine + bleomycin + prednisone; IDAG, idarubicin + cytarabine + granulocyte-colony stimulating factor; VDLP, vincristine + daunorubicin + L-asparaginase + prednisone; ID, idarubicin; ADM, adriamycin; VCR, vincristine.

^a^ Patient was diagnosed with M4. AML treatment stopped intramedullary recurrence for more than one year. HSCT treatment was performed at the end of the first DAC + HAA regimen, and intramedullary recurrence was seen 6 months after transplantation. The second HSCT treatment was performed on August 2018 after the remission of chemotherapy with the DAC + HAA regimen.

^b^ Oral chidamide during treatment.

^c^ Patient was diagnosed with M5. The first relapse occurred with central nervous system leukemia, and she was not treated with HSCT after the remission of DAC + HAD chemotherapy. Intramedullary relapse occurred 32 months after treatment remission, and the patient died after receiving DAC + HAA chemotherapy once.

^d^ Patient was diagnosed with M2 with *AML1-ETO* and had multiple bone marrow relapses before coming to our hospital.

**Table 3:** Response to treatment modalities.

	CR (*n* = 25)	NR (*n* = 12)	*P*-value
Age (%)			0.740
<10 years	18 (72.0)	8 (66.7)	
≥10 years	7 (28.0)	4 (33.3)	
Gender (%)			0.414
M	11(44.0)	7 (58.3)	
F	14 (56.0)	5 (41.7)	
WHO classification subtype (%)			0.655
M1	2(8.0)	0	
M2	9 (36.0)	4 (33.3)	
M3	0	0	
M4	3 (12.0)	1 (8.3)	
M5	8 (32.0)	4 (33.3)	
M6	1 (4.0)	0	
M7	2 (8.0)	3 (25.0)	
Genetic anomaly at diagnosis (%)			0.326
Normal karyotype without any molecular abnormality	8 (21.6)	5 (13.5)	
*t* (8 : 21)	5 (20.0)	1 (8.3)	
*AML1-ETO*^a^	7 (18.9)	1 (2.7)	
*Mixed lineage leukemia-AF9*	1 (2.7)	0	
*WT1-ABL*	1 (2.7)	2 (5.4)	
*CEBPA*	1 (2.7)	1 (2.7)	
*FLT-ITD*	2 (5.4)	0	
*CBFβ/MYH11*	1 (2.7)	1 (2.7)	
*BCR-ABL*	0	1 (2.7)	
*TET2*^b^	0	2 (5.4)	
*EVI1*	1 (2.7)	0	
*NRAS*	1 (2.7)	0	
*NuP98/KDM5A*	1 (2.7)	0	
Disease status (%)			0.367
Relapsed	20(80.0)	11 (91.7)	
Refractory	5 (20.0)	1 (8.3)	
Recurrence time (%)^c^			0.499
≤18 months	21(95.5)	8 (88.9)	
>18 months	1 (4.5)	1 (11.1)	

CR, complete remission; NR, non-remission.

^a^ All patients with *t* (8 : 21) chromosome abnormalities tested positive for the *AML1-ETO* gene.

^b^ One patient tested positive for the double mutation of the *CEBPA* gene.

^c^ The total number of relapses was 31.

Twenty-three patients (62.2%) underwent HSCT and four did not achieve CR. Some children with bone marrow cytology also did not achieve CR and still opted for HSCT treatment. Three patients had multiple relapses.

### Complications

The complications suffered by the patients are summarized in Table 4. Bone marrow suppression was the most common complication after chemotherapy. During the period of bone marrow suppression, the chances of the development of bacteria, viruses, and fungi infection increased. WBC, ANC, hemoglobin concentration, and platelet count were included in the statistical data recorded after chemotherapy. A total of 33 patients in this study developed grade III–IV bone marrow suppression after treatment, including 8 with grade IV bone marrow suppression.

**Table 4:** Complications resulting from HDS treatment.

	CR (*n* = 25)	NR (*n* = 12)	*P*-value
WBC, ×10^9^/L (%)			0.075
<1.3	14 (73.7)	18 (94.7)	
≥1.3	5 (26.3)	1 (5.3)	
ANC, ×10^9^/L (%)			0.146
<1	17(89.5)	19 (100)	
≥1	2 (10.5)	0	
Hemoglobin concentration, g/L (%)			0.290
<80	16(84.2)	18 (94.7)	
≥80	3 (15.8)	1 (5.3)	
Platelet count, ×10^9^/L (%)			0.676
<50	15(78.9)	16 (84.2)	
≥50	4 (20.1)	3 (15.8)	
Infected during 60 days after treatment (%)			0.635
CMV viremia	2(10.5)	2 (10.5)	
Adenovirus	1 (5.3)	0	
Bacteria infection	6 (31.6)	9 (47.4)	
Fungus infection	4 (20.1)	3 (15.8)	
Liver function abnormalities (%)	6(24.0)	3(25.0)	0.947
Kidney damage (%)	0	0	–
Multiple relapses	1 (4.0)	2 (16.6)	–

WBC, white blood cell; ANC, absolute neutrophils count; CMV, cytomegalovirus; CR, complete remission; NR, non-remission.

Within 60 days after treatment, the most common infection occurring in patients with fever during granulocytopenia is pneumonia, which is mostly caused by bacteria, irrespective of whether CR in incidences of bloodstream infection (BSI) is similar. Prompt improvement of immune reconstitution may reduce the infection rate. In this study, during chemotherapy, nine patients developed reversible liver function abnormalities, six of these nine achieved CR, and no patient suffered kidney damage. Some non-hematological adverse reactions such as nausea, vomiting, diarrhea, mucositis, and constipation were resolved after treatment.

### Survival

**Figure 2:**
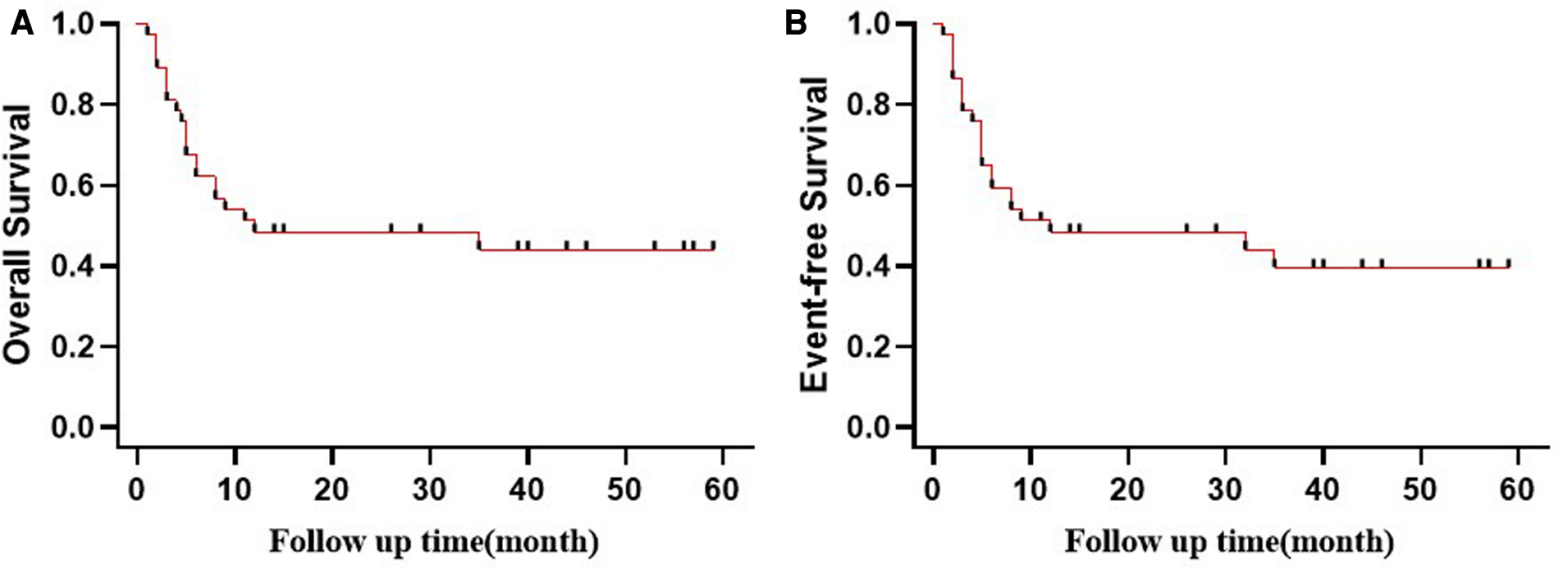
Probability of (**A**) OS and (**B**) EFS (Kaplan–Meier method) for patients stratified by treatment.

By August 31 2022, the median duration of follow-up was 11 months (range, 1–59 months). Seventeen patients survived. Unfortunately, 20 of the relapsed patients (54.1%) died. Seven patients died of myelosuppression after transplantation. A large majority of these deaths occurred because of myelosuppression infection, which was the leading cause of death. Events related to infection include pneumonia, sepsis, or disease progression. Figure 2 shows the survival rates of all patients, whose 3-year OS and EFS rates were 45.9% and 43.2%, respectively.

## Discussion

The prognosis of rel/ref AML remains poor, with only a minority of patients responding to intensive salvage therapy. An important childhood AML study reported that approximately 5% of AML children suffer from refractory diseases and 30% experience relapse (19). In recent years, an increasing number of methods have been used in the treatment of rel/ref AML; however, the remission rates range only from 18% to 55% (20–22). In China, the treatment of children with rel/ref AML usually includes a combination of classical anthracyclines such as daunorubicin (DNR) and cytarabine (Ara-c) (23). However, in choosing this standard chemotherapy, our team found that this combination was poorly tolerated and produced serious side effects such as a high infection rate (100%) and organ dysfunction. The use of anthracyclines also increases the risk of early cardiac toxicity. Therefore, our team has been seeking a safer and more effective chemotherapy for rel/ref AML. Since 2010, we have been following the chemotherapy regimen of 5-aza-2-deoxycytidine (DAC) combined with homoharringtonine + cytarabine + aclarubicin (HAA regimen) (12), which initially helped achieve satisfactory therapeutic effects (13). However, with the gradual increase in the number of cases treated, we found that the remission rate of this chemotherapy regimen for recurrent and refractory AML after transplantation gradually decreased. With improvements in the genome database and culture technique of the primary tumor cell, the selection of antitumor drugs based on drug sensitivity and resistance is gaining more acceptance by many hospitals (15). Since 2017, in our department, some patients with rel/ref AML have been opting for HDS treatment, in order to seek a more individualized treatment plan. In this report, an improved outcome of HDS for pediatric patients was seen. The CR rate of HDS treatment was 67.6%, which is significantly higher than that of conventional treatment (21, 24). We found that infection occurring after chemotherapy is the main factor leading to the decline in the survival rate.

In this study, most of the deaths were found to be infection-related, including infection/sepsis. There is growing evidence of an increased association between certain pathogens and the susceptibility to infection. Many drugs are widely used in disease processes, usually in combination, which makes it difficult to determine a link with the risk of infection (25). Therefore, anti-infection treatment becomes particularly important to the whole treatment process of rel/ref AML. One of the biggest obstacles to AML treatment in developing countries is the high infection rates of multidrug-resistant microorganisms (26). The prophylactic use of antibiotics is a way to reduce infection-related mortality (27). Therefore, for patients with neutrophils who have not recovered, anti-infection treatment should not be ignored. Carvalho AS et al. reported the relationship between the recovery of neutrophils and infection in the myelosuppression phase of chemotherapy (28); Gram-negative bacterial infections constituted a majority of infections (26). We counted the number of WBC and ANC in the myelosuppression phase of each patient who underwent HDS treatment. Although there were different degrees of myelosuppression, only eight patients had grade IV myelosuppression. Reducing the number of chemotherapeutic drugs may help reduce the chances of infection. Our team is currently assessing the possibility of implementing preventive antimicrobial treatment, but this work is being hampered by limited financial resources and poor compliance of some patients.

Scholars in various countries have developed a variety of antitumor drug sensitivity prediction tests of different malignant tumor diseases to realize the rationalization of drug use for tumor patients and the individualization of chemotherapy (29). In recent years, with the discovery of increasingly new genes, targeted drugs have been used in the treatment of rel/ref AML (12, 15). This has led to a gradual increase in chemotherapy drugs used by patients. It has been reported that even if the number of chemotherapy drugs in the induction period of acute lymphoblastic leukemia (ALL) patients is appropriately reduced, it has no obvious effect on the induction results, but it can reduce the probability of infection (27). However, it is not clear whether reducing the use of chemotherapy drugs has an impact on the induction of remission in AML patients. Patients who chose HDS treatment can potentially reduce the use of chemotherapy drugs. Our study shows that the HDS treatment remission rate reached 67.6%, which is higher than that of the standard chemotherapy (18%–55%) (21, 24). Most importantly, chemotherapeutic drugs can be used specifically, reducing the chances of drug trial and error, as well as reducing the financial pressure on patients. In the process of the treatment of diseases, we found that the response to treatment and drugs from in vitro experiments showed different results, and the reason behind such variation needs to be further investigated.

Age is a negative factor affecting the prognosis of AML; however, for childhood leukemia, age has no obvious effect on the treatment results. In the AML–Berlin/Frankfurt/Muenster (BFM) studies, age could not be used as an independent prognostic factor for infants and adolescents (19, 30, 31). Unlike adult patients, the daily management of child patients is more dependent on the cooperation of patients' families. However, many of the patients' families lack infection control or proper hygienic practices. Appropriate care support structures impact the survival rates of children, and therefore, these need to be significantly improved in low-income/middle-income countries (32). In our study, it was found that, despite the non-remission of bone marrow, some patients still chose HSCT. This reflected the great desire on the part of the patients’ families for the survival of their children. Our treatment achieved a higher CR rate (67.6%) but did not improve the OS rate (45.9%), and this may be related to the disease management after transplantation.

Data from 20 cancer centers in Egypt showed that the 5-year OS rate was 40% in the absence of uniform national treatment plans (33). The governmental annual healthcare expenditure per capita correlated with the 5-year OS. From this, it is evident that issues regarding service shortage, inadequate support to improve healthcare infrastructure, and poor government spending on healthcare need to be addressed (34). Although some patients achieved CR, they did not choose HSCT. Certain patients did not complete follow-up treatment, possibly due to the fact that their families were not able to afford the cost. Such patients were excluded from our study. Currently, in China, the long treatment process and high cost are still preventing families from receiving complete treatment.

Our study is limited by its retrospective nature and short duration of follow-up. The number of patients in this study is limited, and it is a single-center study. However, due to the rarity of rel/ref AML in children, it was difficult to conduct a prospective randomized controlled trial. As children with this disorder were managed by the same team with supportive care measures, this study still provides comparative evidence of the values of rel/ref AML in children. The occurrence of repeated relapse and the development of clonal diseases need to be further monitored. We hope to carry out clinical studies with a larger number of patients in the future.

In conclusion, the study results suggested that the HDS-guided chemotherapy regimen is associated with a high bone marrow remission rate and a good safety profile in rel/ref AML pediatric patients, and therefore, may be a good bridging treatment for HSCT. This regimen is very important for the treatment of infection in bone marrow suppression after chemotherapy. However, this deserves further confirmation in a large prospective study.

## Data Availability

The original contributions presented in the study are included in the article/Supplementary Material, and further inquiries can be directed to the corresponding author.
